# Building the foundation for a modern patient-partnered infrastructure to study temporomandibular disorders

**DOI:** 10.3389/fdgth.2023.1132446

**Published:** 2023-05-15

**Authors:** Laura Elisabeth Gressler, Terrie Cowley, Marti Velezis, Suvekshya Aryal, Deanne Clare, John W. Kusiak, Allen W. Cowley, Art Sedrakyan, Danica Marinac-Dabic, Michelle Reardon, Lisa Schmidt, Jennifer Ginsburg Feldman, Vincent DiFabio, Suzie Bergman, Vahan Simonyan, Yelena Yesha, Ingrid Vasiliu-Feltes, Justin Durham, Andrew I. Steen, Phillip Woods, Flavia P. Kapos, Nilsa Loyo-Berrios

**Affiliations:** ^1^ Center for Devices and Radiological Health (CDRH), Food and Drug Administration, Silver Spring, MD, United States; ^2^ Division of Pharmaceutical Evaluation and Policy, University of Arkansas for Medical Sciences, Little Rock, AR, United States; ^3^ TMJ Association Milwaukee, WI, United States; ^4^ Department of Population Health Sciences, Weill Cornell Medicine, New York, NY, United States; ^5^ Department of Physiology, Medical College of Wisconsin, Madison, WI, United States; ^6^ Oral and Maxillofacial Surgery, University of Maryland Medical System, Baltimore, MD, United States; ^7^ Dentistry on Officers Row, Vancouver, WA, United States; ^8^ Embleema, Washington, DC, United States; ^9^ Department of Computer Science, University of Miami, Miami, FL, United States; ^10^ SofThread, Miami, FL, United States; ^11^ School of Dental Sciences, Newcastle United Kingdom; ^12^ Newcastle-Upon Tyne Hospitals’ NHS Foundation Trust, Newcastle, United Kingdom; ^13^ Center for Child Health, Behavior and Development, Seattle Children’s Research Institute, Seattle, WA, United States

**Keywords:** temporomandibular joint, temporomandibular joint disorders, temporomandibular joint dysfunction syndrome, temporomandibular joint disc, delphi, coordinated registry network, data infrastructure and integration

## Abstract

**Background:**

Conflicting reports from varying stakeholders related to prognosis and outcomes following placement of temporomandibular joint (TMJ) implants gave rise to the development of the TMJ Patient-Led RoundTable initiative. Following an assessment of the current availability of data, the RoundTable concluded that a strategically Coordinated Registry Network (CRN) is needed to collect and generate accessible data on temporomandibular disorder (TMD) and its care. The aim of this study was therefore to advance the clinical understanding, usage, and adoption of a core minimum dataset for TMD patients as the first foundational step toward building the CRN.

**Methods:**

Candidate data elements were extracted from existing data sources and included in a Delphi survey administered to 92 participants. Data elements receiving less than 75% consensus were dropped. A purposive multi-stakeholder sub-group triangulated the items across patient and clinician-based experience to remove redundancies or duplicate items and reduce the response burden for both patients and clinicians. To reliably collect the identified data elements, the identified core minimum data elements were defined in the context of technical implementation within High-performance Integrated Virtual Environment (HIVE) web-application framework. HIVE was integrated with CHIOS™, an innovative permissioned blockchain platform, to strengthen the provenance of data captured in the registry and drive metadata to record all registry transaction and create a robust consent network.

**Results:**

A total of 59 multi-stakeholder participants responded to the Delphi survey. The completion of the Delphi surveys followed by the application of the required group consensus threshold resulted in the selection of 397 data elements (254 for patient-generated data elements and 143 for clinician generated data elements). The infrastructure development and integration of HIVE and CHIOS™ was completed showing the maintenance of all data transaction information in blockchain, flexible recording of patient consent, data cataloging, and consent validation through smart contracts.

**Conclusion:**

The identified data elements and development of the technological platform establishes a data infrastructure that facilitates the standardization and harmonization of data as well as perform high performance analytics needed to fully leverage the captured patient-generated data, clinical evidence, and other healthcare ecosystem data within the TMJ/TMD-CRN.

## Introduction

Temporomandibular disorders (TMD) encompass a wide variety of conditions, (e.g., developmental, genetic, inflammatory, degenerative, neoplastic, traumatic, metabolic and idiopathic); affect the temporomandibular joint (TMJ) as well as the surrounding muscles, bones, connective tissue, nerves, and vasculature; and can refer to a specific and heterogenous group that includes myalgia, myofascial pain with referral, arthralgia and headache attributed to TMD (1).

TMD is common and affects an estimated 31 percent of adults in the worldwide (2). TMD can cause both pain and functional limitations. The intensity and impact of the pain experienced can vary but substantially impacts people's daily lives including their ability to eat, communicate, and sleep (3–7). Further, TMD is associated with substantial morbidity, affecting quality of life and work productivity. As an example, it is estimated that for every 100 million working adults in the US, TMD contributes to 17.8 million lost workdays annually (8). In the United Kingdom, studies demonstrated that the quality and quantity of the work performed by individuals with TMDs is decreased by 12 percent each. This translates to a “hidden” cost to employers of £584 and £1,225 in lost productivity for each 6-month period among individuals living with TMD (9, 10). The natural history and etiology of the disorder are poorly understood and thus, appropriate treatment options are difficult to determine, limited, and complex (11).

The location of the TMJ in the orofacial region has to date resulted in most related research occurring within the clinical area of dentistry. For these reasons, the majority of TMD research has been directed and funded by the National Institute of Dental and Craniofacial Research (NIDCR) in direct contrast to the current understanding of the body as an interdependent system (12). Furthermore, a large proportion of historical and current treatments for TMD have focused (biomechanically) on the jaw joint, teeth, and affiliated musculature. Existing treatments include, but are not limited to: occlusal adjustments; local injections of steroids or botulinum toxin; various surgical procedures such as joint replacement; as well as more conservative, non-surgical treatments including acupuncture, physiotherapy and behavioral modification (13). There is limited evidence regarding the safety and effectiveness of these treatment options (14). As such, there are no formal guidelines for TMD treatment and management formulated by professional groups in the USA. In lieu of formal guidelines, various organizations have put forth scientific statements, parameters of care, and recommendations (15–18).

Conflicting reports from varying stakeholders related to prognosis and outcomes following placement of TMJ implants, in addition to data from the Food and Drug Administration's (FDA) MedWatch system, and patient accounts shared with the TMJ Association (TMJA) raising concerns regarding the safety and effectiveness, gave rise to the development of the TMJ Patient-Led RoundTable initiative. The TMJ Patient-Led RoundTable comprises key stakeholders as partners including patients, the FDA, NIDCR, the Agency for Health Care Research and Quality (AHRQ), the American Association of Oral and Maxillofacial Surgeons (AAOMS), TMJ implant manufacturers, clinicians, scientists, advocacy organizations, and other experts, all under the auspices of Medical Devices Epidemiology Network (MDEpiNet). More importantly, the RoundTable is a vehicle in which patients play critical roles throughout all related activities. Following its first meetings, the members of the RoundTable assessed the current availability of data and the ability of third parties to access, collect, and compile scientifically valid information related to selected aspects of TMD patient therapies. The RoundTable concluded that a strategically Coordinated Registry Network (CRN) is needed to collect and generate accessible data on TMD and its care that is sufficiently relevant and reliable. This is necessary to better understand the disparate treatment pathways and outcomes that patients experience.

While the importance and need of high quality dental records to inform decision-making has been established (19), there remains significant variance in how TMD patient dental and medical records are captured and recorded (20–22). Previous efforts have attempted to develop and validate the content taxonomy for dentistry to standardize and harmonize patient data (23). The development of a CRN complements and builds upon these efforts given its ability to creatively organize relevant data systems, establish and grow the capacity of existing data sources, harmonize various data sources including medical and dental records, and leverage data sources that are created for other purposes such as documenting and billing for care (24). CRNs allow for the efficient capture of evidence needed to evaluate TMJ/TMD throughout the disease course (25). The development and establishment of CRNs for these purposes have been successful for various disorders the neurovascular and gynecological clinical space (26–29).

The first foundational step toward building a CRN within the TMD clinical was to identify a core minimum dataset to inform the TMJ/TMD-CRN. From that point it becomes possible to establish the data infrastructure that facilitates the standardization and harmonization of data as well as perform high performance analytics needed to fully leverage the captured patient-generated data, clinical evidence, and other healthcare ecosystem data within the TMJ/TMD-CRN. The aim of this study was therefore to advance the clinical understanding, usage, and adoption of a core minimum dataset for TMD patients.

## Methods

### Multistakeholder partnership, data source identification, and data element extraction

The first TMJ Patient-led RoundTable was held on June 16, 2016, at the FDA headquarters in Silver Spring, Maryland. The meeting led to the formation of four working groups that were tasked with addressing specific areas of study and a Steering Committee to oversee the project as a whole. The RoundTable reconvened on May 11, 2018, at the FDA Headquarters to update participants on the results of the working group projects, establish a roadmap to address highlighted gaps in knowledge regarding TMD, and to identify data collection needs for the development of high quality, real-world evidence (RWE).

**Figure 1:**
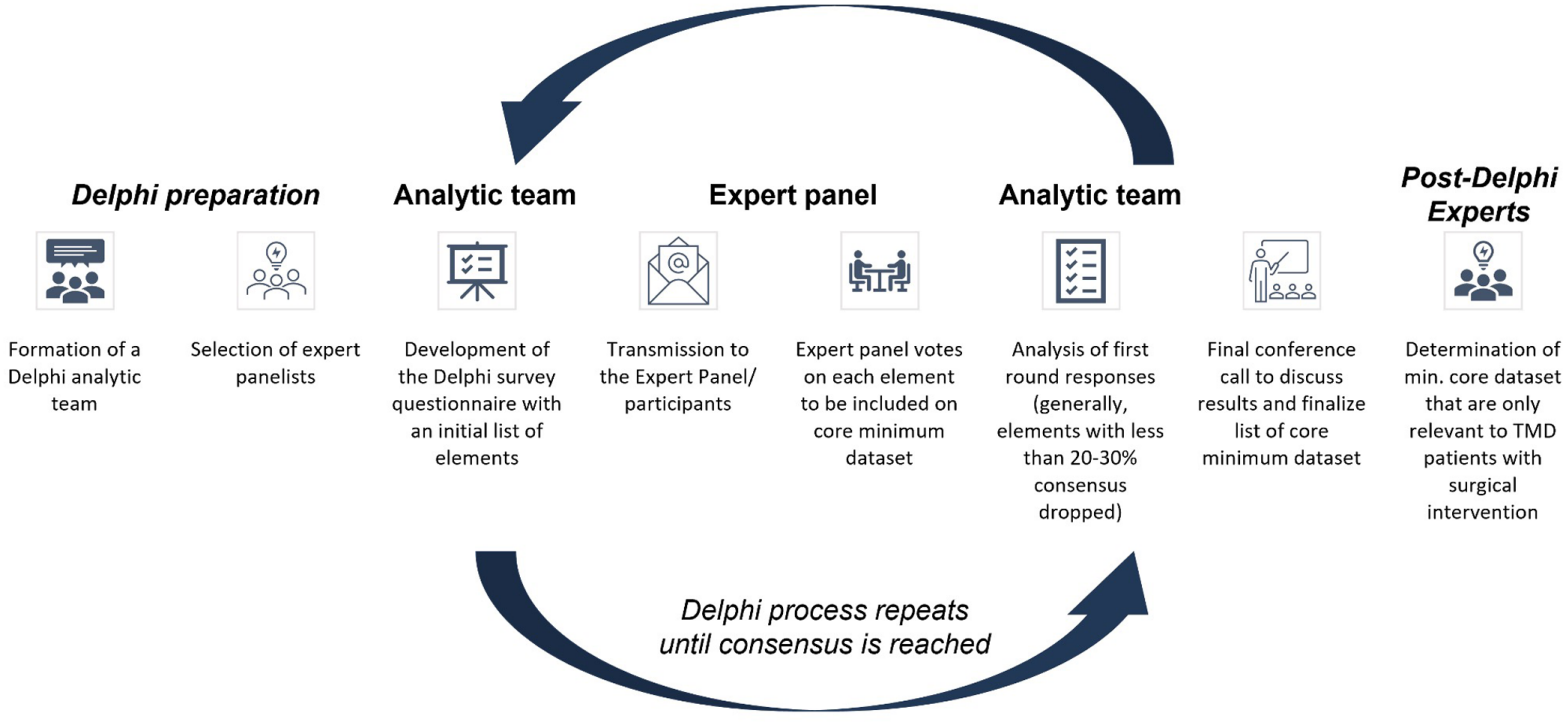
Delphi process.

Findings from the working groups indicated that TMD is a multisystem disorder with complex etiology centered on heightened central nervous system activity that contributes to TMD dysfunction and symptomology (30). For these reasons, many patients experience overlapping comorbidities (8). To comprehensively identify data elements for inclusion in the TMJ/TMD-CRN core minimum dataset for the Delphi process (Figure 1), data elements were extracted from existing data sources that identified conditions overlapping with TMD (31). Additionally, data elements were extracted from published peer-reviewed original research, protocols on clinicaltrials.gov, and existing real-world data sources that aim to evaluate TMD and TMD devices. Finally, data elements in 522 postmarket surveillance (PMS) and premarket studies submitted for 4 different TMJ implants were reviewed and extracted.

The core minimum dataset for TMJ/TMD-CRN was developed with the help of the Delphi method (32). The Delphi method facilitates group decision making with a panel approach by bringing together experts from various backgrounds in a conference or, in this case, an online platform. Delphi can be conducted in a survey format to reach consensus among the identified stakeholders without any group bias generated from group dynamics or face-to-face responses. The Delphi method has been leveraged for consensus building among content taxonomy in patient records within dentistry previously (23).

In total, 531 distinct data elements were captured and extracted from the various sources of published, publicly available, and regulatory data sources. These data sources included 4 premarket studies, 5 postmarket studies (PMS) and 6 real-world data (RWD) sources (33–36), and 1 clinical trial (37). Following the data elements extraction, the informatics and clinical working groups organized the data elements according to their context within the clinical workflow, removed redundancies, and added identified missing data elements to the Delphi process. The informatics and clinical working groups were comprised of patients, patient advocates, clinicians, regulators, researchers, informaticians, as well as representatives of industry and professional societies. The working groups met bi-weekly over the course of 6 months and identified 92 stakeholders for Delphi participation. The identified stakeholders represented various organizations including regulatory bodies, industry, academicians, clinicians, and most importantly patients and patient advocacy groups (Supplementary Appendix Table S1).

Finally, the Delphi survey questionnaire was designed by the MDEpiNet Coordinating Center within Weill Cornell Medicine (WCM) using an online platform, Qualtrics, and distributed to 92 Delphi participants. The survey was analyzed by the WCM team and discussed with the TMD core working group co-chairs via conference calls. The team made a group decision to drop elements that received less than 75% consensus. The consensus percentages were rounded up to the closest number, and all variables achieving more than 75% consensus were finalized as the core minimum dataset for patient-generated and clinical data. Participants were provided with the opportunity to respond with open comments and feedback at the end of the survey. The feedback received was considered by the analysis team to generate recommendations for specific data elements, wherever appropriate. Only one round of the Delphi was conducted as target consensus of 75% was achieved on the first distribution for a majority of the data elements resulting in a finalized core minimum dataset.

Following this process the items reaching 75% were presented to Delphi participants. A purposive multi-stakeholder sub-group was selected from the Delphi participants (*n* = 5 patients, *n* = 4 clinicians, *n* = 4 others including regulators and researchers) to triangulate the items across patient and clinician-based experience to remove redundancies or duplicate items. The group specifically examined the items for any opportunity to reduce response burden for both patient and clinician to maximize the usability of the registry. The process for this triangulation was iterative, qualitative, discursive, and undertaken over 5 one-hour virtual meetings of the sub-group chaired by a clinician from outside of the US (JD). All identified CDE common to existing CRNs, such as demographic variables, were mapped to common standard vocabularies and, where relevant, to the source health information systems standards as specified by the Office of the National Coordinator for Health Information Technology (ONC) United States Core Data for Interoperability (USCDI) (38). CDE that were TMJ-specific and accepted by consensus, will be mapped prior to the implementation of the registry.

#### Establishing the technological platform to support the TMJ/TMD-CRN

For an established CRN to reliably collect accurate dataset, a robust technological platform such as the High-performance Integrated Virtual Environment (HIVE), was leveraged (39). The HIVE infrastructure promotes the sustainability and applicability of the developed CRN by: (1) ensuring compliance with relation to cybersecurity and privacy of data access (40, 41) and availability (42); (2) providing vertical scalability for large data volumes and horizontal scalability for variety of data types; (3) facilitating interoperability by employing standards supported by standard development organizations; and (4) supporting various different data entry modalities as well as vast spectrum of data analyses.

The identified core minimum data elements (Supplementary Appendix Tables S1, S2) were defined in the context of technical implementation within HIVE web-application framework. More specifically, the variables were assigned types (e.g., strings, numbers), constraints (e.g., choice bound to a dictionary, numerical ranges, mappings to ontologically codified values), hierarchical interrelations, visibility, and the order of appearance within questionnaires, etc. These definitions ensure the validity of data entry process to avoid mistakes, logical consistence, completeness of the forms, and generate visual aids that facilitate data entry by optimizing the appearance and ease of entry through responsive web-design concepts. This variable engineering and form development process, though being separate from the core Delphi processes, required close communication between developers of HIVE web-application platform and Delphi participants. The developers imposed important questions not only about clinical relevance of variables, but also the relevance and validity of values that can be recorded for those variables.

HIVE was integrated with CHIOS™, an innovative permissioned blockchain platform, to strengthen the provenance of data captured in the registry and drive metadata to record all registry transaction and create a robust consent network. This integration would allow recording of all registry transactions and create a robust consent framework. The designed target ecosystem would maintain all existing functionality of HIVE registry with added functionality including maintenance of all data transaction information in blockchain, flexible patient consent recordation, data cataloging, and runtime consent validation through smart contracts. For this technical feasibility project, the engineering team built and implemented several client-side Application Programming Interfaces (API) operations using Python and smart contracts for the consent module. The Python wrapper was developed in the team's GitHub organization.

## Results

A total of 59 multi-stakeholder participants responded to the Delphi survey including 30 (51%) participants representing patients or a patient advocacy group and 29 (49%) participants representing a clinician, physician, or researcher (Table 1). The results are presented based on the groupings that were present in the Delphi, patient-generated data elements, clinician-generated data elements and general feedback.

**Table 1:** Delphi participants—round 1.

Participant Group (*n* = 59)	%
Patients/patient representatives	51%
Researchers	22%
Clinicians	10%
Researcher/clinicians	10%
Industry/manufacturer	5%
Regulators	2%

Total number of invitations = 92; Total participants = 59; Response rate 64%.

### Patient-Generated data elements

The completion of the Delphi surveys followed by the application of the required group consensus threshold resulted in the selection of 397 data elements (254 for patient-generated data elements and 143 for clinician generated data elements). The data elements that were presented to the Delphi participants with the consensus percentage are summarized in Supplementary Appendix Table S2. The final list of identified patient-generated data elements in the minimum core dataset are shown in Table 2. Beyond patient demographics, the identified data elements captured patient-reported provider related information, provider contact information, any additional information about care, and their preference regarding data security. More specifically, data security captures the patient's preference on whether to grant or remove the treating clinicians' access to the data provided in the TMJ/TMD-CRN. To comprehensively capture that patient's current health status, the medications the patient is currently using, any currently implanted devices, any allergies, any experienced symptoms, and the primary reasons for seeking care were among the data elements. For patient's medical history, there was consensus on capturing the presence of major chronic conditions and medical conditions that co-exist with TMD. These conditions include cardiovascular, dental, endocrine, otorhinolaryngological, ophthalmological, gastrointestinal, genitourinary, hematologic, infectious, musculoskeletal, neurological, general or systematic, rheumatologic and immunological conditions. Given that TMD is difficult to distinguish and the tremendous overlap in diagnosing a TMJ problem as a specific diagnosis when the overlap can involve TMJ disease, muscular inflammation, CNS pain, biopsychosocial (BSP) issues, and other soft and hard tissue/genetic disorders. In addition, the lack of understanding of the etiology and the symptoms of the disease, data elements capturing whether TMD has previously been misdiagnosed and the symptoms that mimic TMD that may have led to the diagnosis were included. Additionally, the patient's social history, the family history of TMD, and whether the patient had a previous device implanted were data elements for which consensus was reached. The patient's past surgical history including receipt of any TMJ treatment procedures, dental implants, alternative TMJ treatment, receipt of medication for jaw necrosis or clenching and bruxism was captured. There was consensus on collecting relevant post-operative outcomes including open bite, range of motion, infection, device removal, device failure, complications occurring during removal procedure, any change in disability status after procedure and reoperations. Data elements related to long-term follow-up were captured to assess patients lost to follow-up, the reasons for loss to follow-up, and any hospital readmissions.

**Table 2:** Core minimum dataset for patient related information.

Data Class	Data Element
Patient demographics—Common *Clarification of context	Last Name
	First name
	Date of birth
	Sex
	Gender * *Note related to importance of knowing impact of hormone therapy*
	Employment status *Note: the context of changes to employment status due TMD pain and/or treatment outcomes
	Disability status *Note: the context of changes to disability status due TMD pain and/or treatment outcomes
	Race
	Ethnicity
	State of residence
	Country
	E-mail address
*Provider related information—Common*	Capturing provider related information
*Additional information about care—Common*	Capturing additional information about patient care to provider
*Data security—Common*	Granting or removing access to data to provider (Y/N)
*Provider contact—Common*	Provider type
	Provider organization
	Last name
	First name
	Address
	Street number and name
	City
	State
	Zip code
	Phone
	Email address
*Medication—Common*	Usage of the prescription medications, over the counter, supplements, herbals (including cannabis) * Note: Need to address compliance of prescribed medications.
*Medication—Conditional*	Any Cancer Therapeutics (e.g, Chemotherapy or Therapeutics Radiation/Radiotherapy) *Note: Should leverage how this is collected in other registries
*Implanted device—Common*	Unique Device Identifier (UDI) available on the implant card
	Device Type
	Manufacturer
	Brand Name
	Device Model
*Allergies—Common*	Any known common allergies * Note: Rephrase as: “Any known allergies (e.g., metal, medications, seasonal, food, etc.)”
*Reason seeking care—Common*	Reason seeking care captured by clinician (Y/N) * Note: Rephrase as: “Patient's description of the reason they are seeking care”
Symptoms—Common *added during post-Delphi review	Symptom Details—Onset (sudden or gradual)
	Symptom Details—Laterality
	Symptom Details—Severity
	Symptom Details—Duration*
	Symptom Type—Symptoms in the Jaw You would be asked to select one or more the following choices: fatigue in your jaw when talking and/or chewing, stiffness in your jaw, clicking with or without pain, popping, cracking, crepitus grating, squishy/fluid sound, squeaking (TMJ Implant patients only), Eustachian tube dysfunction/ear clicking sounds/fullness in the ear, other
	Symptom Type—Symptoms in the mouth and tongue You would be asked to select one or more the following choices: difficulty opening and closing, pain/difficulty to close mouth, pain/difficulty to open mouth, tongue thrusting, mouth breathing, difficulty swallowing, pain while swallowing, gross motor control, fine motor control, difficulty chewing, dietary restrictions related to chewing, lack of taste, distortion of taste, other
	Symptom Type—Symptoms in the eyes You would be asked to select one or more the following choices: pain behind your eye(s), vision correction, blurry vision, other
	Symptom Type—Symptoms in the ears You would be asked to select one or more the following choices: earaches, fullness or ringing in your ears, Eustachian tube dysfunction, Fluid/drainage from ear, ear tubes, other
	Symptom Type—Headaches You would be asked to select one or more the following choices: Cluster headache, Migraine headache, Sinus headache, Tension headache, Fogginess, other
	Symptom Type—Sleep problem or disorder You would be asked to select one or more the following choices: insomnia (inability to fall asleep), obstructive sleep apnea (airway is blocked), central sleep apnea (airway is not blocked), complex/mixed sleep apnea syndrome, other
	Symptom Type—Symptom Triggers You would be asked to select one or more the following choices: eating, yawning, crying, weather, mask wearing, poor sleep/position, prolonged sitting, talking, posture, coughing/sneezing, stress, dental x-rays, other Medicap procedures/testing, other
*Past medical history—cardiovascular—Common*	Coronary Artery Disease
	Artificial Heart Valve
	Congenital Heart Defect
	Heart Murmur
	High blood pressure
	Low blood pressure
	Infective Endocarditis
	Mitral Valve Prolapse
	Rheumatic Fever
	Abnormal Heart Rhythm
	Raynaud's Phenomenon
	Vasculitis
	Aneurysm
	Postural Orthostatic Tachycardia Syndrome (POTS)
*Past medical history—non-specific—Common*	Headaches
	Chronic fatigue syndrome
*Past dental history—Common*	Tooth Deterioration—missing, damaged/cracked, caries (tooth decay), dry socket, loose, root resorption *Rephrase: “Significant dental health issues (e.g., loose, missing, damaged or cracked teeth, tooth decay, dry socket or root resorption, gum recession, frenulum developed from scar tissue)*
	Failed Dental treatment (e.g., fillings, orthodontia, wisdom teeth removal, crown work, restorations, Grinding down teeth, splints etc.) *Rephrase: “Significant dental procedures (note any complications)”.*
*Past medical history—glands and organ for endocrine conditions—Common*	Adrenal Disorders
	Diabetes
	Thyroid Disorders
	Sexual Dysfunction
	Hormone disorders (e.g., PCOS, Infertility, male hormone disorders, female hormone disorders)
	Menopause
	Painful Menstrual Periods
	Endometriosis
	Premenstrual Syndrome (PMS)
	Estrogen-based Hormone Replacement Therapy (including hormonal birth control)
*Past medical history—Otorhinolaryngology—ENT conditions -Common*	Salivary stone
	Sinusitis
	Clenching and Bruxism
*Past medical history—Ophthalmology and vision acuity*	Past medical history—Ophthalmology and vision acuity (Y/N)
*Past medical history—Gastrointestinal conditions—Common*	Acid Reflux/GERD/Heartburn/hiatal hernia
	Ulcerative Colitis/Crohn's
	Gastritis
	Intestinal/Stomach Ulcers
	Irritable Bowel Syndrome
	Malnutrition, weight fluctuation
	Liver disease/Jaundice/Hepatitis
	Pancreatic disease
*Past medical history—Reproductive and urinary system (Genitourinary conditions)—Common*	Bladder infections/bladder dysfunction/incontinence
	Interstitial Cystitis
	Prostatitis
	Nephroptosis
	Urolithiasis
	Chronic Pyelonephritis
	Testicular Tumors/Disorders
	Vulvar vestibulitis syndrome/vulvodynia
*Past medical history—Hematologic conditions—Common*	Anemia
	Chronic swollen Lymph Nodes
	Hemophilia
	Blood transfusion
Past medical history—Infectious diseases—Common *added during post-Delphi review	Sexually Transmitted Disease
	Lyme/Tick/or insect borne diseases or infections
	MRSA or other chronic infection (staph, strep)
	Arthritis—Infectious
	Epstein–Barr virus (EBV)*
Past medical history—Musculoskeletal conditions—Common *added during post-Delphi review	Osteoporosis
	Metabolic bone disease, bone remodeling
	Muscular Dystrophy
	Osteochondritis dissecans
	Eagle Syndrome
	Fibromyalgia
	Congenital/Craniofacial Disorders (e.g., Hemifacial Microsomia/Goldenhar Syndrome, hyperplasia etc.)
	Bisphosphonate Related Osteonecrosis of the Jaw (BRONJ)
	Medication-related osteonecrosis of the jaw (MRONJ)
	Ehlers-Danlos syndrome (EDS), connective tissue disorder or hypermobile joints/double jointed*
	Avascular Necrosis of temporomandibular joint*
*Past medical history—Neurological conditions—Common*	Eyebrow/Eyelid/Facial paralysis & Numbness
	Bell's Palsy
	Burning Mouth Syndrome, burning tongue
	Cerebral palsy
	Epilepsy/Seizures/Convulsions
	Ernest Syndrome
	First bite syndrome/Frey Syndrome
	Sleep Disorders/Fatigue/Obstructive Sleep Apnea/Central Sleep apnea
	Movement Disorders/Oromandibular dystonia/dystonic tremor/cervical dystonia
	Trigeminal Neuralgia (pain in the nerve)
	Trigeminal Neuropathy (pathology of the nerve—e.g., pain or weakness—can cause neuralgia)
	Vertigo/dizziness/spaciness
	Multiple Sclerosis
	Migraine, Cluster, tension, Premenstrual migraine
	Myasthenia Gravis
	Stroke
	Trigeminocardiac reflex
	Traumatic injury to the head or neck
*Past medical history—General or systemic conditions—Common*	Oncology history (e.g., Cancer of Bone, Breast, GI, Leukemia, Lymphoma, Lung, Prostate, Mandibular, oral)
	Conditions affecting the Lungs/Pulmonary (e.g., asthma)
	Reproductive history/past pregnancy history (e.g., number of pregnancies and live births)
*Past medical history—Immune system (Rheumatology or Immunology)—Common*	Arthritis—Traumatic
	Arthritis—Rheumatoid
	Arthritis—Osteoarthritis/Degenerative
	Arthritis—Psoriatic
	Arthritis—Gouty
	Arthritis—Seronegative
	Mast Cell Activation Syndrome (MCAS)
*Misdiagnosed as TMJ/D or if TMJ/D (mimics)—Common*	Misdiagnosis of TMD (mimics) Y/N * Rephrase as: “Were you experiencing symptoms or diagnosed with another condition (e.g, dental condition, headaches, temporal arteritis, neuropathic conditions, salivary stones, angina, arthritic condition, lyme/tick or insect borne disease or infection), which was later determined to be TMD, or vice versa?
*Patient's social history—Common*	Use of Tobacco/Vaping
	Tobacco/Vaping Usage information (e.g., use tobacco to manage TMD pain, smoke tobacco, chew tobacco, vape, other)
	Use of Alcohol products
	Alcohol usage information (e.g., use alcohol beverages to manage TMD pain, drink once a month, drink once a week, drink multiple times a week, drink daily, other (specify)
	Use of Recreational Drugs
	Recreational Drugs Usage information (e.g., use recreational drugs to manage TMJ pain, use once a month, use once a week, use multiple times a week, daily use)
*Family history of TMD—Common*	Family history of TMJ/D (Y/N)
*Patient's previous device implant/treatment—Previous TMJ Treatment—Common*	Consultation with a medical or dental specialist for TMJ/D
	Type of specialist
	Information about the specialist (e.g., name or providers)
	Pre TMJ implant information about the patient's TMJ condition prior to TMJ implant
	Post TMJ implant information about the patient's TMJ condition after a TMJ implant
	Previous TMJ/TMD Treatment
	Time from symptom onset to diagnosis of previous Treatment
*Previous TMJ Injection/Medication—Common*	Steroid injections
	PRP injections
	Prolotherapy/Trigger Point injections/nerve blocks
	Botox Injections
	Iontophoresis with Dexamethasone, lidocaine, benzocaine, septocaine and/or others
*Previous TMJ Therapy—Common*	Massage Therapy
	Cranial Sacral Therapy
	Myofascial & Precision Neuromuscular massage therapy
	Therapeutic exercises (posture and mechanical training)
	Manual Therapy
*Previous Alternative Treatment—Common*	Acupuncture/Dry needling
	Myofunctional/Speech Therapy
	Physical Therapy/Physiotherapy
	Chiropractic Treatment
	IV Ozone
	Shock wave therapy
	Hyperbaric oxygen therapy
	Cold laser therapy
	Ultrasound therapy
	Magnetic therapy
	Heat therapy
	Breath Work
	Physical Rehabilitation
*Previous TMJ Education/Counseling/Training—Common*	Splint/orthotic/mouth guard/night guard (Custom vs. OTC)
	Education/Counseling/Training Behavioral Therapy/Counseling (biofeedback, CBT, Relaxation training, hypnosis, stress management, mindfulness)
*Past Dental History—Conditional*	Full mouth restorations
*Past surgical history—TMJ surgical procedure—Common*	Arthrocentesis
	Arthroplasty
	Arthroscopy
	Orthognathic surgery
	Total Joint Replacement (TJR)
	Joint Replacement for Tumor, Trauma, Others with Vascular/Bone Fibula Grafts
	Intraoral Vertical Ramus Osteotomy And Intermaxillary Fixation
*Past surgical history—TMJ intervention*	Date of previous TMD Surgical procedures
	Type of previous TMD Surgical procedures
	Previously failed surgeries * Rephrase as: Surgery or procedure addressed or fixed the patient's problem (if not, provide additional information)
	Number/type of surgical treatments (to be calculated by the entries)
*Past surgical history—TMJ treatment procedure*	Autogenous Reconstruction
	Condylectomy/Condylotomy (spacer used)
	Discectomy/Reconstruction/Repair
	Disc replacement (material, Silastic, fat, temporalis flap)
*Past surgical history—dental—Common*	Failed non-surgical procedure * Rephrase as: “Non-surgical management for your TMD aggravate your condition
	Wisdom tooth extraction complication
*Past surgical history—dental—Conditional*	Type of Dental Implant
	Dental Implant Removed
	Dental implant or restoration complication
*Past surgical history—alternative TMJ treatment—Common*	Myofascial & Precision Neuromuscular treatment, muscular electrical stimulation (TENS)
	Manipulation of mandible *Rephrase as: “Other forms of treatment, not stated elsewhere. Specify)
*Patient's medication list—jaw necrosis—Common*	Brand name
	Generic name
	Drug class
*Patient's medication list—clenching and bruxism—Common*	Brand name
	Generic name
	Drug class
*Descriptive diagnosis of patient visit—Common*	Descriptive diagnosis of patient visit captured by clinician * Rephrase as: “Describe what you have been told is causing your signs and symptoms/problems?”
*Post-operative outcomes—Conditional*	Open Bite
	Range of Motion (ROM)
	Crossbite
	Overjet
	Deviated Opening
	Device integration with bone or soft tissue
	Infection e.g., biofilm infection, others?
	Device Removal (including details about the device or surgical site)
	Device Failure (including damage to device or device component)
	Complications occurring during removal procedure
	Change in disability status after procedure * Rephrase as: “Following your treatment for TMD have you become unable to maintain employment?” Possibly date-referenced when felt unable to maintain employment
	Reoperations
*Longitudinal follow-up—Common*	Lost to Follow-up and Lost to follow-up Type
	Readmission and Date

The data classes below have been grouped as “*Common*” or “*Conditional*” based on the post-Delphi discussions of how to address data elements that may only relate to a subpopulation of patients e.g., patients undergoing Total Joint Replacement, which are marked as conditional in the table below and all others relate to all patients.

### Clinician-generated data elements

Data elements collected by clinicians that are included in the minimum core dataset are summarized in Table 3. The clinician-generated data elements that were presented to the Delphi participants with the consensus percentage are summarized in Supplementary Appendix Table S3. These data elements included clinician information, findings from a physical exam, exams related to TMJ (e.g., jaw function/dysfunction, pain onset, pain duration, Wilkes staging classification for internal derangement, Angle's classification), clinical assessment (e.g., malocclusion, deviated opening laterality, maximum interincisal opening, range of motion), and whether these assessments led to the diagnosis of TMD.

**Table 3:** Core minimum dataset for clinician collected information.

Data Class	Data Element
*Physical exam—Common*	Height
	Weight
*Physical exam—Conditional*	BMI
*TMJ assessment—Common*	Jaw Function/Dysfunction * Consider the following assessment tool: JFLS-SF
	Diet
	Pain onset
	Pain quality
	Pain duration
	Angle's Classification * Note: For specific indications. This should be automated.
*TMJ assessment—Conditional*	Wilkes Staging Classification for Internal Derangement * * Note: Optional for specialist clinicians only
	Charlson comorbidity Index * Note: To reduce burden of additional questions, automation may pull values for available conditions from past medical history*. This is a ten-year mortality predictor.*
*Clinical assessment—exams—Common*	Malocclusion * Note: Revise to “occlusion” with the following response options: class 1,2 (div 1 or 2), 3 and AOB, lateral open bite, scissor bite. This can be incorporated in the Angle's classification.
	Deviated opening laterality, mm from midline * Note: Allow options of deviation or no deviation, and laterality instead of measure in mm from midline.
	General condition of dentition (e.g., tooth wear, decay, etc.) * Note: Expand the value set to include: Tooth wear or cracked/fractured/broken (Incisal, Cuspid, Bicuspid, Molars)
	Maximum Interincisal Opening * Note: Include additional information: With Pain, Without Pain, Assisted or Unassisted
	Range of Motion (ROM) * Note: Include Excursive Movement
	Facial asymmetry * Note: Include the following response options: temporalis, masseter hypertrophy and atrophy, chin point deviation, other/specify
	Other, specify^a^
*Clinical assessment—exams—Conditional*	Examination of temporalis tendon
	Pressure pain threshold * Note: Consolidate to one item about muscles of mastication tenderness on palpation that is familiar to patient's complaint
*Clinical assessment—diagnosis—Common*	Diagnosis based on clinical assessment (Y/N)
*Clinician information—Common*	Clinician Identifier
	Type of Clinician
	Facility Identifier
Laboratory findings—Common *revised tests during post-dephi review	C-Reactive Protein (CRP) Test
	Complete Blood Count (CBC) Test
	Rheumatoid Factor (RF) Test
	Erythrocyte Sedimentation Rate (ESR) Test*
	Immunofluorescent ANA with reflex ENA testing*
	Anti-CCP antibodies*
*Imaging findings—Common*	CT Scan with or without contrast
	Magnetic Resonance Imaging (MRI) findings
	Panoramic findings
	Other Imaging (identify)^b^
*Clinical assessment—TMJ/D specific diagnostics tests—Common*	Clinical assessment—TMJ/D specific diagnostics tests (Y)^c^
*TMD treatment procedures—Conditional* *(if procedure was completed)*	Date of Procedure
	Procedure Code
	Interventional Site (body location)
	Procedure Status (e.g., Completed, Treatment Aborted, Incomplete)
	Procedure Urgency (e.g., Elective, Urgent, Emergency)
	Facility (location)
	Surgeon(s) performing the procedure
	Length of Procedure
*Arthrocentesis procedures—Conditional* *(if procedure was completed)*	Anesthesia Type
	Device Details—Single Needle Gauge
	Device Details—Double Needle Gauge
	Device Details—Small Diameter Arthroscopy (1MM) Type
	Procedure Details—Complications
	Procedure Details—Additional Procedures Needed
*Arthroscopy procedures—Conditional* *(if procedure was completed)*	Anesthesia Type
	Single Portal
	Double Portal
	Triple Portal
	Level 1
	Level 2
	Level 3
	Biopsy Required
	Fluid Analysis
	Lavage Type
	Lavage Amount
	Medication Type
	Medication Amount
	Complications
	Use of Laser
	Additional Procedures Needed
*Arthroplasty procedures—Conditional* *(if procedure was completed)*	Anesthesia Type
	Prep and Drape
	Incision Type
	Soft Tissue Debridement
	Disc Displacement Reduction And Fixation
	Meniscectomy
	Hard Tissue Reduction And Contouring: Condyle
	Hard Tissue Reduction And Contouring Fossa/Eminence
	Temporalis Flap Interposition
	Fat Graft Interposition Group this and 1 above together under autografting to joint space?
	Spacer Synthetic Temporary Material joint
	Complications
	Additional Procedures Needed
*TMJ total joint replacement (TJR) procedures—Conditional* *(if procedure was completed)*	Anesthesia
	Prep And Drape
	UDI Stock TMJ TJR
	UDI Custom TMJ TJR
	Incisions Type
	Incisions Position * Note: Combine with Incision Type
	Fat Graft
	Bone Cement
	Fossa Component/Screws
	Mandible Component/Screws
	Complications
	Operation Changed From Custom To Stock TJR
	Additional Procedures Needed
*Coronoidectomy procedures—Conditional* *(if procedure was completed))*	Anesthesia
	Prep And Drape
	Extra Oral
	Intra-Oral
	With TJR
	Removal Of Coronoid Process In Toto
	Leave Part Of The Coronoid Process Attached To Temporalis Muscle
	Complications
	Additional Procedures Needed
*Orthognathic surgery with TMJ TJR procedures—Conditional* *(if procedure was completed)*	Anesthesia
	Prep And Drape
	Maxilla Surgery
	Mandible Surgery And Splints
	Maxilla And Mandible Surgery
	Turbinectomies
	Total Joint Replacement
	Complications: Bleeding, Control Of Bleeding
	Additional Procedures Needed
*Joint replacement for tumor, trauma, others with vascular/bone fibula grafts procedures—Conditional* *(if procedure was completed)*	Anesthesia
	Prep And Drape
	Resection Of Tumor Or Bony Fracture Per Protocol
	Harvest Of Vascular/Bony Fibula Graft
	Reconstruction Of TMJ/Fossa/Mandible With Fibula Graft
	Fibula Grafts Complications
	Additional Procedures Needed
*Intraoral Vertical Ramus Osteotomy And Intermaxillary Fixation procedures—Conditional* *(if procedure was completed)*	Anesthesia
	Prep And Drape
	Intermaxillary Fixation
	Complications: Bleeding, Control Of Bleeding, Other
	Additional Procedures Needed
*TMD device details and device characteristics—Conditional* *(if procedure was completed)*	Device UDI
	Device type
	Device class
	DI number
	Company Name
	Brand Name
	Model Number
	Implant material
*TMD medications—Conditional* *(As prescribed by treating physician pre/post-surgery)*	Dose
	Dose Units
	Code
	Type Class
	Start Date
	End Date
*Additional Post-operative outcomes—Conditional*	Chronic lymphocytic infiltrate present
*Additional longitudinal follow-up elements—Common*	Mortality—Date of death
	Mortality—Cause of death
*Patient survey tools for TMJ/TMD patients—Common*	EuroQoL five dimension (EQ-5D-5l) (Pre-Op to 60 months)
	Jaw Function limitation scale 8—(Pre-Op, 3 months, 12 months)
	OHIP-TMD (Pre-Op to 60 months)
	Pain Numeric Rating Scale (NRS) (Pre-Op to 60 months)
	SF-12 (Pre-Op, 3 months, 12 months) *Note: These were also evaluated by the TMD PROMs Working Group and will be addressed in their recommendation*

^a^ Associated responses are included in the results section.

^b^ ibid.

^c^ ibid.

There was consensus on including specific data elements from any laboratory or imaging obtained prior to a potential TMD diagnosis. These included Antinuclear Antibodies tests, C-Reactive Protein tests, Complete Blood Count tests, Rheumatoid Factor tests, Rheumatological Lab tests (e.g., anti-citrullinated protein antibody test), CT scan with or without contrast, Magnetic Resonance Imaging findings, and Panoramic findings. The participants reached consensus on minimal data elements for all procedures, including the date of the procedure, procedure code, intervention site, procedure status, procedure urgency and length of procedure. Procedure related elements were also captured for arthrocentesis, arthroscopy, arthroplasty, TMJ total joint replacement, coronoidectomy, orthognathic surgery with TMJ total joint replacement, joint replacement for tumor, trauma, others with vascular/bone fibula grafts, and intraoral vertical ramus osteotomy and intermaxillary fixation procedures. If a TMJ implant was implanted then device-related characteristics to be captured were identified such as the unique device identifier (UDI) and the associated data (e.g., the device type, device class, model number, and implant material). Any TMD medications including the dose, dose units, type class, start date, and end date are also to be recorded. Data elements related to post-operative outcomes included the presence of chronic lymphocytic infiltrate and longitudinal follow-up elements included mortality. Finally patient survey tools were identified as crucial elements for the core minimum dataset. The patient survey tools included overall quality of life patient reported outcome measures (PROMs), psychosocial status and TMD symptom specific patient reported outcomes (PROs). This included the EuroQoL five dimension (43), Jaw Function limitation scale (44), Oral Health Impact Profile for Temporomandibular Disorders (OHIP-TMD) (45), Pain Numeric Rating Scale (46), and Short Form-12 (47). Patients will be able to complete the varying PROMs at different timepoints throughout their TMJ/TMD disease course. The TMJ/TMD-CRN will need a flexible data infrastructure to capture any existing or future validated PROMs.

In addition, there were specific concepts that allowed for open comments and/or recommendations for capturing additional elements of clinical information. First, for clinical assessment with exams, 78% participants commented to capture details such as pre-auricular area and external auditory canal (EAC) palpation for pain; pain triggers; pain alleviation; previous facial/head/neck trauma; incisal/occlusal wear of teeth; TMJ palpation; and occlusal contacts in maximum intercuspation. In terms of laboratory findings, although these did not reach the consensus cut-off, it is important to note that 63% participants recommended that there should be additional captures but didn't provide any suggestions. For imaging findings, 44% recommended that there should be additional captures, and at least one person commented that bone scan and simple radiographs should be captured. In terms of clinical assessment with diagnostics tests, 50% participants responded affirmative to capturing diagnostic-related information, and provided the following elements or use of the assessment tools: Beighton score, Research Diagnostic Criteria (RDC)-TMD^1^, TMJ ankylosis specific quality of life (QoL) questionnaire, pain on loading, bone scan, clinical exam and imaging, Mahan test, tongue bite test for synovitis, diagnostic criteria for TMD, pain on lateral palpation at the condyle, and response to diagnostic anesthetic block.

### General feedback on the survey

Based on the general feedback responses at the end of the survey, the comments generally reiterated the recommendations for additional data elements, identified concerns or suggested limitations of the survey. The recommendations for additional data elements were further reviewed by the working group members from the perspective of achieving optimal balance between the granularity and least burdensome approach.

#### Triangulation of items by purposively sampled subgroup of delphi participants

The group agreed on the majority (85%) of items in the patient data collection (Table 2). Major points of discussion involved the inclusion of past medical history, past dental history procedures, mimics, and changes in disability status after the procedures. Past medical history includes the determination of history or presentation of symptoms (e.g., Headaches, Clenching and Bruxism), use of medications (e.g., compliance with prescribed medications, receipt of chemotherapy and/or radiotherapy), as well as metal and medication allergies. Past dental history procedures include but are not limited to full mouth restorations, extractions, orthodontics, and their associated outcomes. Discussion ensued around the aforementioned items given their potential for removal following the Delphi process. The group resolved however to retain these items.

Four items, in Table 2, were consolidated due to the redundancy. These were: “currently prescribed medications…”, as it could be covered by another item; “any chemotherapy” as it was merged with another item; two items on metal and medication allergies as it was covered by the broader item on “any known…allergies”. The section (18 items) on mimics for the TMD was simplified to one question thereby removing 17 items and was rephrased carefully—“Were you experiencing symptoms or diagnosed with another condition, which was later determined to be TMD, or vice versa?”—in order to capture the bidirectional nature of a mimic. That is TMD could mimic some other disorder or vice versa and both are important to be captured. Similarly, the section on past dental history was simplified to two items—“Significant dental health issues” and “Significant dental procedures” rather than four due to identified item redundancy.

The group noted and suggested a solution to the lack of a validated variable in the patient data entry relating to the patient's diagnosis of TMD. The suggested solution was to request completion of the 6 item TMD screener validated by Gonzalez et al. as part of the data capture (48). The patients and clinicians in the subgroup emphasized that this screener did not act as a gatekeeper to inputting the remaining data in the registry, but rather served as a validated item to increase utility of analyzing the data produced from the registry.

In the clinician data collection (Table 3) the group also agreed on the majority of items (95%). Two items were identified as being conditionally required in the minimum core dataset: the Wilkes Staging Classification for Internal Derangement and examination of the temporalis tendon. Five items were revised to prevent redundancy and increase utility of the registry. The items included: (1) Medications that may cause jaw necrosis, and clenching and bruxism as they may change over time and are better kept to analysis of the medication list, (2) Utility of clinical assessment tools e.g., Charlson Comorbidity Index, (3) Inclusion of the findings from clinical assessment exams, (4) Laboratory findings that were not specific enough (and underwent additional enumeration with clinical stakeholders during the post Delphi review), and (5) Patient reported outcomes after operation as they may be captured by PROMs and/or in the clinician generated data elements.

Several identified data elements are shared across other CRNs within other clinical spaces. The format, data type, response options and mapping to the current terminology standards specified by ONC's US Core Data for Interoperability (USCDI) and shown in Table 4.

**Table 4:** Coordinated registry network shared data Elements and terminology mappings.

Common Data Elements	Format (Datatype)	Response Options	USCDI v3 Mappings^1^
Sex	Code (string) and Code System (uri)	• Female • Male • Asked but Unknown • Other	SNOMED International, Systematized Nomenclature of Medicine Clinical Terms (SNOMED CT®) U.S. Edition, March 2022 Release
Gender	Code (string) and Code System (uri)	• Female • Male • Female-to-Male (FTM)/Transgender Male/Trans Man • Male-to-Female (MTF)/Transgender Female/Trans Woman • Identifies as non-conforming gender • Additional gender category or other • Choose not to disclose	SNOMED International, Systematized Nomenclature of Medicine Clinical Terms (SNOMED CT®) U.S. Edition, March 2022 Release
Race	Code (string) and Code System (uri)	• American Indian or Alaskan Native Asian Black or African American Native Hawaiian or other Pacific Islander White More than one race Asked but Unknown • Other	The Office of Management and Budget Standards for Maintaining, Collecting, and Presenting Federal Data on Race and Ethnicity, Statistical Policy Directive No. 15, as revised, October 30, 1997^2^
Ethnicity	Code (string) and Code System (uri)	• Hispanic or Latino Not Hispanic or Latino • Asked but Unknown • Other	The Office of Management and Budget Standards for Maintaining, Collecting, and Presenting Federal Data on Race and Ethnicity, Statistical Policy Directive No. 15, as revised, October 30, 1997^3^

^1^ https://www.healthit.gov/isa/united-states-core-data-interoperability-uscdi

^2^ See proposed OMB updates. https://www.federalregister.gov/documents/2023/01/27/2023-01635/initial-proposals-for-updating-ombs-race-and-ethnicity-statistical-standards

^3^ *ibid*

#### Generated technological platform

**Figure 2:**
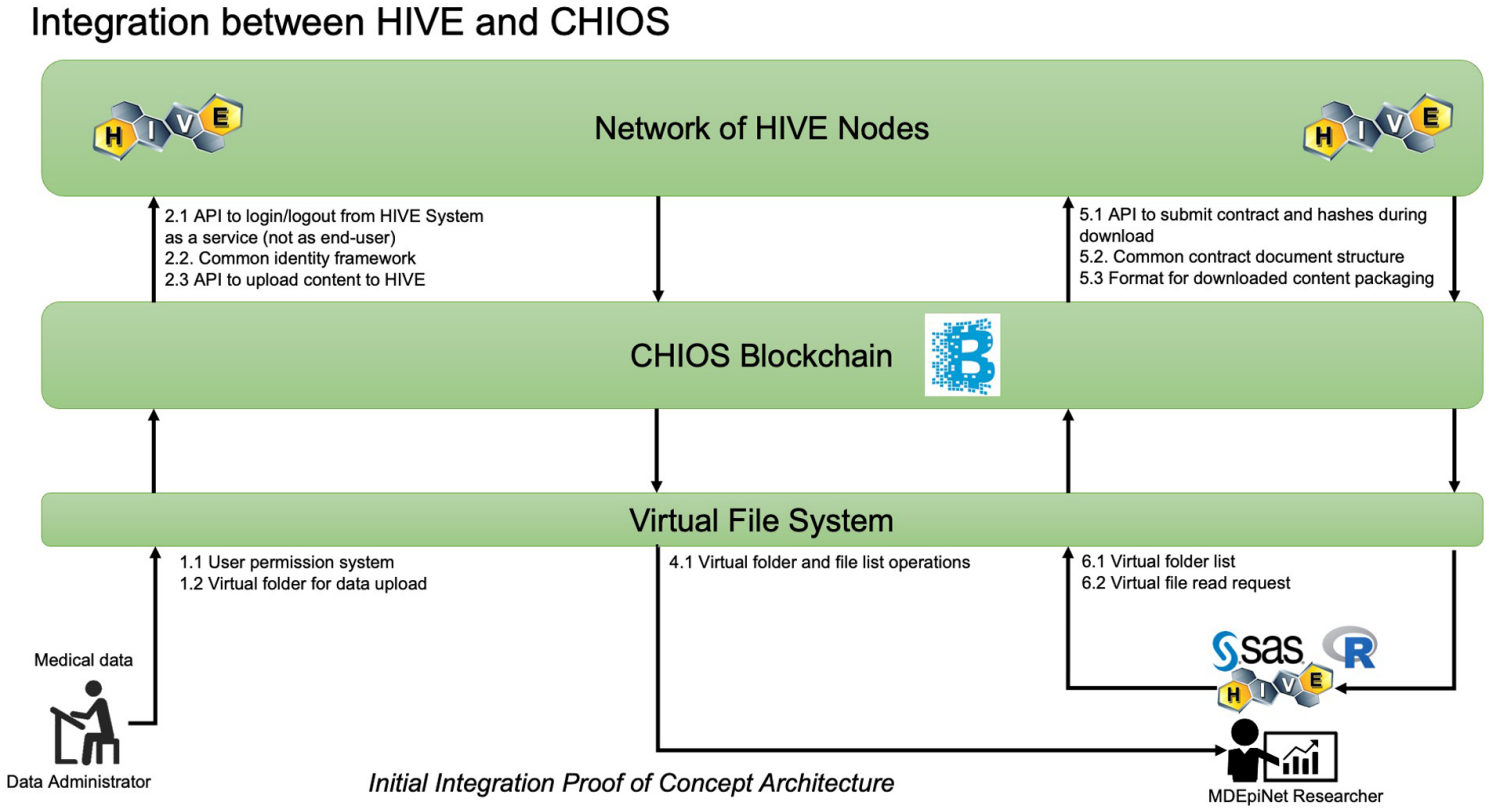
Integration of High-performance Integrated Virtual Environment (HIVE) and CHIOS™.

**Figure 3:**
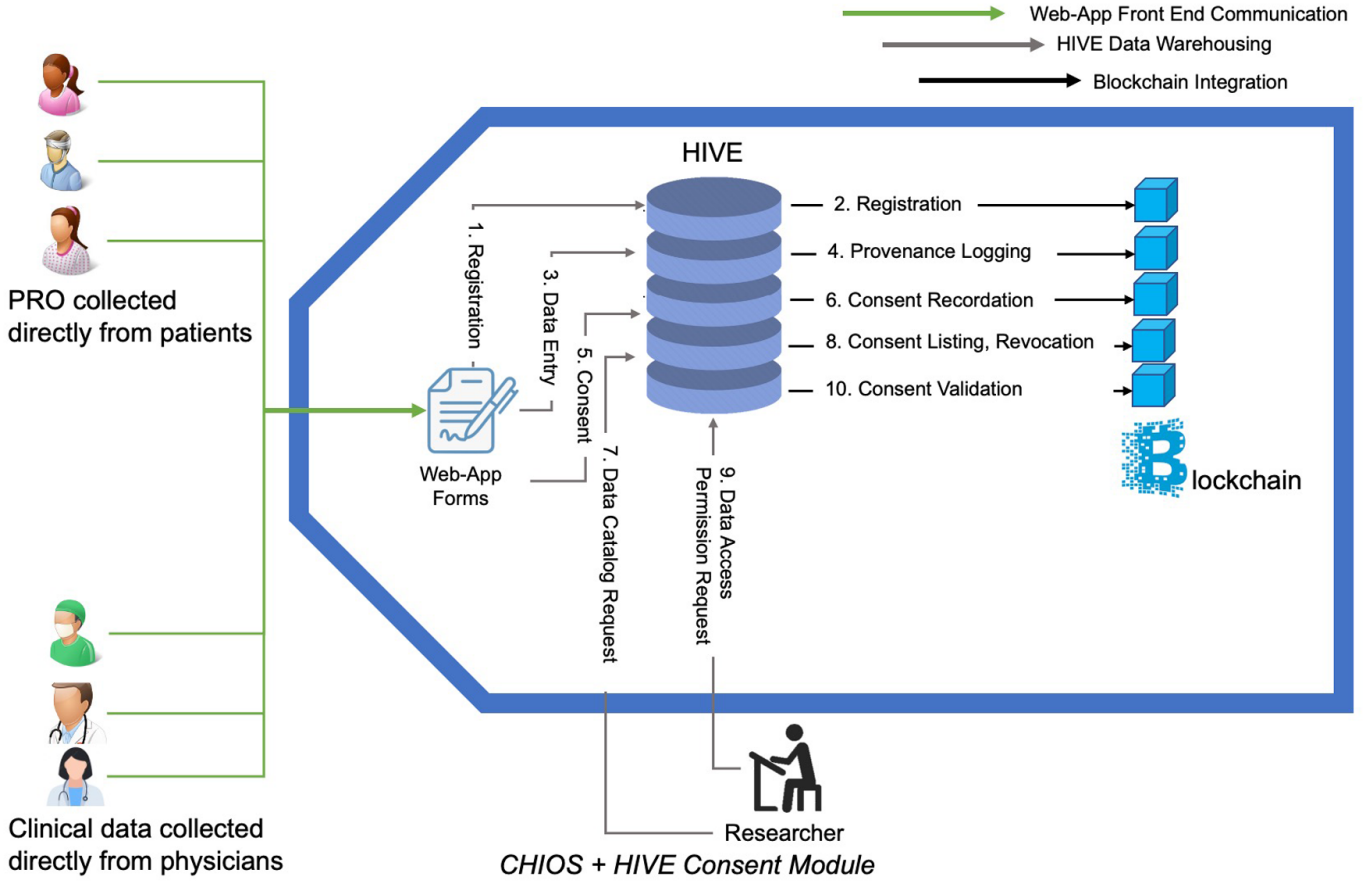
Use of smart contact to collect, securely store, and leverage collected data within High-performance Integrated Virtual Environment (HIVE). Steps outlined within the CHIOS™-HIVE Consent Module: (1) **Registration:** Patients and doctors register on HIVE driven registry web-app portal. (2) **Registration:** HIVE registers in blockchain on user’s behalf. (3) **Data entry:** Users enter information in Web-App. (4) **Provenance logging:** HIVE records the transaction metadata on Blockchain. Information on who, when, which data-type and which fields have been entered will be transmitted to Blockchain via a smart contract. Actual values of the entered fields will not be transmitted. (5) **Consent:** Patients create and sign a consent form on a web-app allowing particular end users/researchers/doctors access their data. (6) **Consent recordation:** The signed consents are translated into harmonized constructs and transferred to the blockchain via a smart contract. (7) **Data cataloging:** Researcher queries on what type of data are available from how many patients in order to understand the landscape of data availability. (8) **Consent listing, revocation:** patient can list the existing consents they have previously provided; they are given opportunity to revoke consents. (9) **Data access permission request**: Doctor or researcher requests to see the patients data. (10) **Consent validation:** HIVE submits request to smart contract on the blockchain to validate consent between list of patients and requestor. Decision is made on allowing the requestor to retrieve data based on the smart contract execution outcome. **Transaction history (not shown in diagram):** HIVE can request the list of all transaction metadata from blockchain layer for auditing and monitoring purposes.

The developers established permissioned blockchain for storing data to enhance data security scenarios. The integration proof of concept architecture is depicted in Figure 2. The use of a permissioned blockchain for storing transaction hash data allows for enhanced data security scenarios. Every data operation in HIVE was verified for its authenticity by an immutable record in the blockchain along with a set of requisite access control permissions associated with the originator of the operation. To demonstrate the CHIOS™ and HIVE integration, the team built out a simple API client for HIVE since one did not exist. The team was able to successfully implement a handful of client side API operations using Python. The CHIOS Consent Module effort consisted of both an API and a smart contract that were developed by the Softhread team to record HIVE user registration and consent operations on the blockchain. This metadata, as committed to the blockchain, is used to verify whether a user has consented to a set of data operations to the untrusted parties that may be requesting that user's data from HIVE. Both metadata provenance and non-repudiation were of focus. The first time ever integration of HIVE and CHIOS™ within the consent module addressed metadata provenance and non-repudiation, thus, enhancing the fidelity and integrity of the user data consent operations within the HIVE environment. The infrastructure development was completed and demonstrated in early January of 2021 to the TMJ/TMD-CRN showing the maintenance of all data transaction information in blockchain, flexible recording of patient consent, data cataloging, and consent validation through smart contracts. The high-level architecture of this consent module is shown in Figure 3. The developed ecosystem allows patients and clinicians to interact with the web-app to register patient and doctor accounts, enter data fields on a web-app provided pages, and request recorded datasets.

## Discussion

The core minimum dataset, identified through stakeholder and patient engagement, outlines the pre-defined standardized data elements needed to assess TMD treatments and devices. These data elements may be entered by patients, by clinicians, or through a hybrid approach based on available technology. They provide the needed foundation of potential data linkages that will allow for the comprehensive assessment of TMD devices as well as strengthen patients' role in generating real-world evidence including epidemiological surveillance data. The potential data linkages may save time and cost of analyses by leveraging all available information for the different available real-world data sources (e.g., electronic health records, health information systems, registries). The comprehensive assessment of the condition and related treatments using these data sources may be leveraged to inform treatment guidelines, clinical decision making, and regulatory processes such as premarket approvals and postmarket surveillance. The resulting real-world evidence may inform the selection of optimal TMD treatment regimens on an individual basis and the prediction of possible clinical outcomes. It is important to note, however, the presence of equally important data source that informs the final decision-making process— the voices of the patients themselves, who are experiencing symptoms that are not typically captured in clinical studies.

Traditional consensus approaches have chances of bias due to the lack of anonymity and the potential of one person having a large effect on the decision-making process. Delphi is conducted in a series of surveys sent out to stakeholders to collect responses anonymously and individually. The participants are able to provide their honest opinions with suggestions, which are then reviewed and analyzed by the core team. The questionnaires can then be revised and re-distributed to participants for several cycles until target consensus is reached (49, 50). The Delphi process allowed for the patients' voice to be amplified while simultaneously engaging multiple other relevant stakeholders, including clinicians, researchers, manufacturers, and FDA, with varying perspectives and experiences with TMD and their associated treatments. Despite the varying perspectives, consensus was achieved. Of note, patients played a substantial role in leading the initial identification and extraction of data elements. Patients also made up more than half of the respondents within the Delphi process. This allowed for the identification of elements that are vital in understanding the patient journey including symptoms and complementary or alternative treatment options that are not necessarily captured or supported in other clinical evidence. The organization and execution of the Delphi process enabled the enhancement of the clinical workflow model by organizing the data elements in logical groupings that allow for the seamless integration of information into the reported data. These groupings include patient reported symptoms organized by body system, past medical history, clinical evaluation, management, treatment outcomes, as well as PROMS. It is important to note that some identified data elements overlap with the survey items included in the identified relevant PROMs^2^. There was significant concurrence between the working groups with respect to the proposed patient survey and assessment tools identified for TMJ/TMD, and the final recommendations will be issued by the TMD PROMS Working Group.

Several limitations associated with the Delphi process should be noted. Even if consensus was achieved, the results may not represent the priorities of all stakeholders. For example, some respondents noted that the representation of the data elements did not align well with the various stages in the natural progression of the disease. More specifically, some elements are related to the pre-diagnosis phase of TMD while other data elements address complications of surgery in more advanced post-diagnosis phases of TMD. Others emphasized the importance of capturing progression from simple to advanced conditions, temporary complaints vs. chronic symptoms, symptoms prior to diagnosis and following a TMD diagnosis, or receipt of non-surgical treatment vs. surgical treatment. Additional comments expressed by the respondents include that: (1) data elements may not be targeted to the clinicians based on their clinical specialties; (2) the data elements were not always based on current clinical evidence and medical concepts; (3) failed treatment or misdiagnosis was not clearly explained; (4) distinctions need to be made between overlapping conditions with the TMD compared to coexisting conditions not related to the TMD; and (5) refinement of the terminologies (e.g., therapies vs. treatment/management) should be incorporated during the next phase of pilot testing of the core minimum data set capture. The refinement of terminologies will be crucial given that the current core data elements are meant to encompass a wide variety of TMD cases. An additional module identifying data elements, such as complexities with regards to laboratory testing, imaging, and procedure-related characteristics, that are more specific to subpopulations with more severe forms of TMD that require, for example, total joint replacement may be necessary to comprehensively capture the experiences of this population. The data elements are meant to encompass TMD as a whole, however, data elements specific to subpopulations with more severe forms of TMD may be needed. Despite these limitations, it is important to note the strengths of the study. Strengths of our methodology include the use of an iterative approach and the incorporation of perspectives from varying stakeholder groups, especially patients, who played a crucial and significant role throughout the entirety of the study. All participants equally influenced the refinement of the core minimum dataset. The anonymous fielding of the survey decreases the potential bias associated with group dynamics in a face-to-face setting and the final triangulation provided by the sub-group review means the item sets can be considered robust and valid representations of both patient and clinician perspectives. Additional strengths associated with the methodology employed include the meaningful engagement and crucial role of various stakeholders including patients, clinicians, professional societies, researchers, manufacturers, and the FDA. The involvement and collaboration of these stakeholders in the various working groups as well as their participation in the Delphi survey ensured that the identified CDE are comprehensiveness and relevant. The participants' future feedback following the initiation of data collection will help future collaborative work to better align and strategically streamline evidence generation needs for all the stakeholders. Furthermore, reviewing existing data sources and studies ensure that many identified elements are already captured in existing data sources which facilitates data linkage and a greater level of interoperability within the establish CRN. Finally, the efforts through the Delphi and the working groups to reduce the number of CDE, in turn, decreases the burden on respondents, supports continuous engagement for longitudinal data input, and enhances user-centricity while still providing valuable information.

The identification of CDEs is an important step prior to data collection and linkage of existing data sources within a CRN. This data collection and linkage of these real-world data sources (RWD) to generate real world evidence (RWE) will improve data monitoring supporting the quicker identification of clinical concerns, potential avoid poor patient outcomes, and thereby inform clinical- and regulatory- decision-making.

The integration of the technological platform for the TMJ/TMD-CRN provides opportunity for unparalleled data entry transaction traceability and data access auditability. All secure transactions such as logging in, entering data values, sharing data access are tracked in the blockchain system in the form of immutable transaction chains. The use of a blockchain smart contract layer allows for the validation of secure data access attempts not only with relation to those who have access to the various data forms, but also control access within usage context: which data fields can be used with which algorithms in which projects within which timeframe and by whom. Additionally, the API can be used as a foundation for future development tasks that would be needed for more extensive integrations of technological platforms that allow for optimized data integrity and enhanced data provenance which is essential in standardization efforts.

It is important to note that HIVE framework allows continuous evolution of data aggregation subsystems. Longitudinal data collection and analysis may in the future reveal minor issues in the initial vision and variable sets such as lack of a relevant variable or ambiguity of a definition. HIVE can dynamically adapt to such issues: (1) it allows adding new variables to existing registry; (2) novel ontological definitions can be incorporated; (3) customizations can be provided as per institution of entry; and (4) patient categories can be introduced that customize data entry modalities. Powerful semantic mapping rule-based engine in HIVE allows to maintain consistency of datasets in evolving registry network without interruptions of live production ecosystem without backward compatibility issues that usually haunt many other registry systems. This dynamic adaptability is foundational to create long-term, longitudinal data ecosystems that can evolve over time.

In conclusion, the data elements identified through the Delphi process represent items that are currently and routinely captured as part of the clinical record as well as the additional patient-centered items that more comprehensively encompass the patients' experience throughout their journey with TMD.

The next step of this project will be to collect these identified CDE through a national infrastructure that will serve as repository for unbiased and high-quality data on TMJ/TMD devices, treatments, and disease course. The development and advancement of this CRN as a robust source of real-world data that leads to the generation of high-quality real-world evidence addresses current strategic priorities of the FDA and existing legislation (51, 52). The modern, state of the art, national infrastructure for data capture will serve as the needed foundation of the TMJ/TMD-CRN and the accrual of high-quality data on TMD and their treatments in the context of a multi-purpose CRN.

## Data Availability

The original contributions presented in the study are included in the article/**Supplementary Material**, further inquiries can be directed to the corresponding author.

## Footnotes

^1^Note that this may be replaced by DC/TMD assessment tool [Eric Schiffman et al. *J Oral Facial Pain Headache*. 2014; 28(1): 6–27.]

^2^Additional PROMs related analysis was done by another TMD working group and will be leveraged in the final representation of the data elements.
