# Supplementary material for: Building the foundation for a modern patient-partnered infrastructure to study temporomandibular disorders
Source: Front Digit Health. 2023 May 15;5:1132446. doi: 10.3389/fdgth.2023.1132446 (PMC10226081; doi:10.3389/fdgth.2023.1132446)
Supplement: Supplementary file 1 [file Table1.docx]

**Appendix**

**Appendix Table 1. Working group members’ background**

| **Category/Background** | **N** |
| --- | --- |
| Patient /Patient Advocate | 7 |
| Clinician | 7 |
| Regulator | 12 |
| Researcher | 13 |
| Informatician | 3 |
| Industry | 2 |
| Professional Society Representative | 4 |

**Appendix Table 2: Consensus for data elements related to patient information**

| **Data Class** | **Data elements** | **Consensus** |
| --- | --- | --- |
| ***Patient demographics*** | Last Name | 81% |
|  | First name | 76% |
|  | Middle name or initial | 54% |
|  | Maiden name | 42% |
|  | Date of birth | 97% |
|  | Sex | 83% |
|  | Gender | 80% |
|  | Employment status | 75% |
|  | Disability status | 81% |
|  | Marital status | 68% |
|  | Race | 78% |
|  | Ethnicity | 76% |
|  | Address – Street number and name | 63% |
|  | Street – second line | 54% |
|  | City | 73% |
|  | State of residence | 78% |
|  | Zip code | 73% |
|  | Country | 86% |
|  | Mobile phone | 69% |
|  | E-mail address | 83% |
| ***Provider related information*** | Capturing provider related information | 98% |
| ***Additional information about care*** | Capturing additional information about patient care to provider | 90% |
| ***Data security*** | Capturing granting or removing access of patient data to provider | 86% |
| ***Provider contact*** | Provider type | 97% |
|  | Provider organization | 86% |
|  | Last name | 95% |
|  | First name | 92% |
|  | Middle initial | 66% |
|  | Address | 78% |
|  | Street number and name | 75% |
|  | Street – second line | 71% |
|  | City | 86% |
|  | State | 88% |
|  | Zip code | 80% |
|  | Phone | 83% |
|  | E-mail address | 92% |
| ***Medication*** | Currently prescribed medications, over the counter, supplements, herbals | 100% |
|  | Usage of the prescription medications, over the counter, supplements, herbals | 97% |
|  | Any Chemotherapy received | 93% |
|  | Any Therapeutics Radiation/Radiotherapy received | 95% |
| ***Implanted device*** | Unique Device Identifier (UDI) available on the implant card | 90% |
|  | Device Type | 100% |
|  | Manufacturer | 98% |
|  | Brand Name | 93% |
|  | Device Model | 97% |
| ***Allergies*** | Metal allergies | 100% |
|  | Medication allergies | 98% |
|  | Any known common allergies | 98% |
| ***Reason seeking care (Y/N)*** | Patient’s reason for seeking care | 100% |
| ***Symptoms*** | Symptom Details – Onset (sudden or gradual) | 100% |
|  | Symptom Details – Laterality | 98% |
|  | Symptom Details – Severity | 98% |
|  | Symptom Type – Symptoms in the Jaw  You would be asked to select one or more the following choices: fatigue in your jaw when talking and/or chewing, stiffness in your jaw, clicking with or without pain, popping, cracking, crepitus grating, squishy/fluid sound, squeaking (TMJ Implant patients only), Eustachian tube dysfunction/ear clicking sounds/fullness in the ear, other” | 97% |
|  | Symptom Type – Symptoms in the mouth and tongue You would be asked to select one or more the following choices: difficulty opening and closing, pain/difficulty to close mouth, pain/difficulty to open mouth, tongue thrusting, mouth breathing, difficulty swallowing, pain while swallowing, gross motor control, fine motor control, difficulty chewing, dietary restrictions related to chewing, lack of taste, distortion of taste, other. | 95% |
|  | Symptom Type – Symptoms in the eyes You would be asked to select one or more the following choices: pain behind your eye(s), vision correction, blurry vision, other | 93% |
|  | Symptom Type – Symptoms in the ears You would be asked to select one or more the following choices: earaches, fullness or ringing in your ears, Eustachian tube dysfunction, Fluid/drainage from ear, ear tubes, other | 95% |
|  | Symptom Type- Headaches You would be asked to select one or more the following choices: Cluster headache, Migraine headache, Sinus headache, Tension headache, Fogginess, other | 97% |
|  | Symptom Type – Sleep problem or disorder You would be asked to select one or more the following choices: insomnia (inability to fall asleep), obstructive sleep apnea (airway is blocked), central sleep apnea (airway is not blocked), complex/mixed sleep apnea syndrome, other | 95% |
|  | Symptom Type – Symptom Triggers You would be asked to select one or more the following choices: eating, yawning, crying, weather, mask wearing, poor sleep/position, prolonged sitting, talking, posture, coughing/sneezing, stress, dental x-rays, other Medicap procedures/testing, other | 95% |
| ***Past medical history – cardiovascular*** | Coronary Artery Disease | 76% |
|  | Artificial Heart Valve | 75% |
|  | Congenital Heart Defect | 75% |
|  | Heart Murmur | 73% |
|  | High blood pressure | 86% |
|  | Low blood pressure | 83% |
|  | Infective Endocarditis | 75% |
|  | Mitral Valve Prolapse | 78% |
|  | Rheumatic Fever | 76% |
|  | Abnormal Heart Rhythm | 75% |
|  | Raynaud’s Phenomenon | 80% |
|  | Vasculitis | 80% |
|  | Aneurysm | 71% |
|  | Postural Orthostatic Tachycardia Syndrome (POTS) | 80% |
| ***Past medical history – non-specific*** | Headaches | 98% |
|  | Chronic fatigue syndrome | 90% |
| ***Past medical history – dental*** | Tooth Deterioration – missing, damaged/cracked, caries (tooth decay), dry socket, loose, root resorption | 92% |
|  | Failed Dental treatment (e.g., fillings, orthodontia, wisdom teeth removal, crown work, restorations, Grinding down teeth, splints etc.) | 90% |
|  | Frenulum developed from scar tissue | 71% |
|  | Gum Recession | 81% |
| ***Past medical history – glands and organ for endocrine conditions*** | Adrenal Disorders | 88% |
|  | Diabetes | 88% |
|  | Thyroid Disorders | 92% |
|  | Sexual Dysfunction | 68% |
|  | Hormone disorders (e.g., PCOS, Infertility, male hormone disorders, female hormone disorders) | 76% |
|  | Menopause | 73% |
|  | Painful Menstrual Periods | 73% |
|  | Endometriosis | 75% |
|  | Premenstrual Syndrome (PMS) | 69% |
|  | Estrogen-based Hormone Replacement Therapy (including hormonal birth control) | 80% |
| ***Past medical history - Otorhinolaryngology - ENT conditions*** | Clenching and Bruxism | 95% |
|  | Salivary stone | 73% |
|  | Sinusitis | 90% |
| ***Past medical history - Ophthalmology and vision acuity (Y/N)*** | Past medical history of ophthalmology and vision acuity | 58% |
| ***Past medical history - Gastrointestinal conditional*** | Acid Reflux/GERD/Heartburn/hiatal hernia | 78% |
|  | Ulcerative Colitis/ Crohn’s | 76% |
|  | Gastritis | 69% |
|  | Intestinal/Stomach Ulcers | 73% |
|  | Irritable Bowel Syndrome | 83% |
|  | Malnutrition, weight fluctuation | 83% |
|  | Liver disease/Jaundice/Hepatitis | 69% |
|  | Pancreatic disease | 63% |
| ***Past medical history - Reproductive and urinary system (Genitourinary conditions)*** | Bladder infections/ bladder dysfunction/incontinence | 66% |
|  | Interstitial Cystitis | 76% |
|  | Prostatitis | 59% |
|  | Nephroptosis | 56% |
|  | Urolithiasis | 56% |
|  | Chronic Pyelonephritis | 56% |
|  | Testicular Tumors/Disorders | 58% |
|  | Vulvar vestibulitis syndrome/vulvodynia | 68% |
| ***Past medical history - Hematologic conditions*** | Anemia | 85% |
|  | Chronic swollen Lymph Nodes | 88% |
|  | Hemophilia | 75% |
|  | Blood transfusion | 68% |
| ***Past medical history - Infectious diseases*** | Sexually Transmitted Disease | 66% |
|  | Lyme/Tick/or insect borne diseases or infections | 85% |
|  | MRSA or other chronic infection (staph, strep) | 81% |
|  | Arthritis - Infectious | 92% |
| ***Past medical history - Musculoskeletal conditions*** | Osteoporosis | 97% |
|  | Metabolic bone disease, bone remodeling | 92% |
|  | Muscular Dystrophy | 85% |
|  | Osteochondritis dissecans | 83% |
|  | Eagle Syndrome | 81% |
|  | Fibromyalgia | 95% |
|  | Congenital/Craniofacial Disorders (e.g., Hemifacial Microsomia/Goldenhar Syndrome, hyperplasia etc.) | 97% |
|  | Bisphosphonate Related Osteonecrosis of the Jaw (BRONJ) | 92% |
|  | Medication-related osteonecrosis of the jaw (MRONJ) | 93% |
| ***Past medical history - Neurological conditions*** | Eyebrow/Eyelid/Facial paralysis & Numbness | 86% |
|  | Bell's Palsy | 88% |
|  | Burning Mouth Syndrome, burning tongue | 90% |
|  | Cerebral palsy | 75% |
|  | Epilepsy/Seizures/Convulsions | 78% |
|  | Ernest Syndrome | 78% |
|  | First bite syndrome/ Frey Syndrome | 88% |
|  | Sleep Disorders/Fatigue /Obstructive Sleep Apnea/ Central Sleep apnea | 98% |
|  | Movement Disorders/ Oromandibular dystonia/dystonic tremor/cervical dystonia | 92% |
|  | Trigeminal Neuralgia (pain in the nerve) | 97% |
|  | Trigeminal Neuropathy (pathology of the nerve - e.g., pain or weakness - can cause neuralgia) | 98% |
|  | Vertigo/ dizziness/ spaciness | 95% |
|  | Multiple Sclerosis | 83% |
|  | Migraine, Cluster, tension, Premenstrual migraine | 92% |
|  | Myasthenia Gravis | 81% |
|  | Stroke | 83% |
|  | Trigeminocardiac reflex | 78% |
|  | Traumatic injury to the head or neck | 97% |
| ***Past medical history - General or systemic conditions*** | Oncology history (e.g., Cancer of Bone, Breast, GI, Leukemia, Lymphoma, Lung, Prostate, Mandibular, oral) | 92% |
|  | Conditions affecting the Lungs/Pulmonary (e.g., asthma) | 75% |
|  | Reproductive history/past pregnancy history (e.g., number of pregnancies and live births) | 68% |
| ***Past medical history – Immune system (Rheumatology or Immunology)*** | Arthritis – Traumatic | 93% |
|  | Arthritis – Rheumatoid | 98% |
|  | Arthritis – Osteoarthritis/Degenerative | 95% |
|  | Arthritis – Psoriatic | 95% |
|  | Arthritis – Gouty | 93% |
|  | Arthritis – Seronegative | 92% |
|  | Mast Cell Activation Syndrome (MCAS) | 92% |
| ***Past medical history – Congenital Craniofacial syndrome (Y/N)*** | Past medical history of congenital craniofacial syndrome | 97% |
| ***Misdiagnosed as TMJ/D or if TMJ/D (mimics) Y/N)*** | Misdiagnosis of TMJ/D or if TMJ/D | 92% |
| ***Mimics*** | Dental condition misdiagnosed as TMJD | 88% |
|  | TMJD misdiagnosed as a dental condition | 90% |
|  | Headaches misdiagnosed as TMJD | 86% |
|  | TMJD misdiagnosed as headaches | 88% |
|  | Temporal arteritis misdiagnosed as TMJD | 83% |
|  | TMJD misdiagnosed as temporal arteritis | 85% |
|  | Neuropathic conditions misdiagnosed as TMJD | 85% |
|  | TMJD misdiagnosed as neuropathic conditions | 85% |
|  | Salivary stones misdiagnosed as TMJD | 83% |
|  | TMJD misdiagnosed as salivary stones | 85% |
|  | Sinusitis misdiagnosed as TMJD | 85% |
|  | TMJD misdiagnosed as sinusitis | 85% |
|  | Angina misdiagnosed as TMJD | 83% |
|  | TMJD misdiagnosed as Angina | 85% |
|  | An arthritic condition misdiagnosed as TMJD | 85% |
|  | TMJD misdiagnosed as arthritic conditions | 86% |
|  | Lyme/tick or insect borne diseases or infection misdiagnosed as TMJD | 85% |
|  | TMJD misdiagnosed as Lyme/tick or insect borne diseases or infection | 85% |
| ***Patients social (use) history*** | Use of Tobacco/ Vaping | 85% |
|  | Tobacco/ Vaping Usage information (e.g., use tobacco to manage TMJ pain, smoke tobacco, chew tobacco, vape, other) | 85% |
|  | Use of Alcohol products | 85% |
|  | Alcohol usage information (e.g., use alcohol beverages to manage TMJ pain, drink once a month, drink once a week, drink multiple times a week, drink daily, other (specify) | 86% |
|  | Use of Recreational Drugs | 83% |
|  | Recreational Drugs Usage information (e.g., use recreational drugs to manage TMJ pain, use once a month, use once a week, use multiple times a week, daily use) | 92% |
| ***Family history of TMJ/D (Y/N)*** | Family history of TMJ/D | 93% |
| ***Patient's previous device implant/treatment*** | Consultation with a medical or dental specialist for TMJ/D | 92% |
|  | Type of specialist | 92% |
|  | Information about the specialist (e.g., name or providers) | 88% |
|  | Pre TMJ implant information about the patient’s TMJ condition prior to TMJ implant | 93% |
|  | Post TMJ implant information about the patient’s TMJ condition after a TMJ implant | 93% |
|  | Previous TMJ/D Treatment | 97% |
|  | Time from symptom onset to diagnosis of previous Treatment | 90% |
|  | Steroid injections | 93% |
|  | PRP injections | 92% |
|  | Prolotherapy/ Trigger Point injections/nerve blocks | 90% |
|  | Botox Injections | 92% |
|  | Iontophoresis with Dexamethasone, lidocaine, benzocaine, septocaine and/or others | 92% |
|  | Massage Therapy | 93% |
|  | Cranial Sacral Therapy | 88% |
|  | Myofascial & Precision Neuromuscular massage therapy | 90% |
|  | Therapeutic exercises (posture and mechanical training) | 90% |
|  | Manual Therapy | 90% |
|  | Acupuncture/Dry needling | 88% |
|  | Myofunctional/Speech Therapy | 88% |
|  | Physical Therapy/Physiotherapy | 92% |
|  | Chiropractic Treatment | 88% |
|  | IV Ozone | 81% |
|  | Shock wave therapy | 81% |
|  | Hyperbaric oxygen therapy | 83% |
|  | Cold laser therapy | 85% |
|  | Ultrasound therapy | 85% |
|  | Magnetic therapy | 83% |
|  | Heat therapy | 88% |
|  | Breath Work | 85% |
|  | Physical Rehabilitation | 88% |
|  | Splint/ orthotic/ mouth guard/ night guard (Custom vs OTC) | 93% |
|  | Education/Counseling/Training Behavioral Therapy/Counseling (biofeedback, CBT, Relaxation training, hypnosis, stress management, mindfulness) | 92% |
| ***Past surgical history – TMJ surgical procedure*** | Full mouth restorations | 90% |
|  | Arthrocentesis | 97% |
|  | Arthroplasty | 97% |
|  | Arthroscopy | 97% |
|  | Orthognathic surgery | 97% |
|  | Total Joint Replacement (TJR) | 98% |
|  | Joint Replacement for Tumor, Trauma, Others with Vascular/Bone Fibula Grafts | 98% |
|  | Intraoral Vertical Ramus Osteotomy And Intermaxillary Fixation | 98% |
| ***Past surgical history – TMJ intervention*** | Date of previous TMD Surgical procedures | 98% |
|  | Type of previous TMD Surgical procedures | 98% |
|  | Previously failed surgeries | 97% |
|  | Number/type of surgical treatments (to be calculated by the entries) | 97% |
| ***Past surgical history – TMJ treatment procedure*** | Autogenous Reconstruction | 95% |
|  | Condylectomy/ Condylotomy (spacer used) | 95% |
|  | Discectomy/Reconstruction/Repair | 95% |
|  | Disc replacement (material, Silastic, fat, temporalis flap) | 97% |
| ***Past surgical history – dental implants*** | Type of Dental Implant | 83% |
|  | Dental Implant Removed | 83% |
|  | Dental implant or restoration complication | 86% |
|  | Failed non-surgical procedure | 86% |
|  | Wisdom tooth extraction complication | 85% |
| ***Past surgical history – alternative TMJ treatment*** | Myofascial & Precision Neuromuscular treatment, muscular electrical stimulation (TENS) | 83% |
|  | Manipulation of mandible | 86% |
| ***Patient’s medication list – jaw necrosis*** | Brand name | 90% |
|  | Generic name | 90% |
|  | Drug class | 86% |
| ***Patient’s medication list – clenching and bruxism*** | Brand name | 90% |
|  | Generic name | 90% |
|  | Drug class | 86% |
| ***Descriptive diagnosis of patient visit captured by clinician (Y/N)*** | Descriptive diagnosis of patient visit captured by clinician | 95% |
| ***Post-operative outcomes*** | Open Bite | 97% |
|  | Range of Motion (ROM) | 97% |
|  | Crossbite | 88% |
|  | Overjet | 90% |
|  | Deviated Opening | 93% |
|  | Device integration with bone or soft tissue | 93% |
|  | Infection e.g., biofilm infection, others? | 95% |
|  | Device Removal (including details about the device or surgical site) | 95% |
|  | Device Failure (including damage to device or device component) | 93% |
|  | Complications occurring during removal procedure | 93% |
|  | Change in disability status after procedure | 88% |
|  | Reoperations | 92% |
| ***Longitudinal follow-up*** | Lost to Follow-up and Lost to follow-up Type | 92% |
|  | Readmission and Date | 86% |

*Note: shaded cells represent data elements with less than 75% consensus; 59 total participants (30 patients and 29 clinicians/researcher)*

**Appendix Table 3. Consensus for data elements related to clinical information**

| **Data Class** | ***Data Elements*** | ***Consensus*** |
| --- | --- | --- |
| ***Physical exam*** | Height | 91% |
|  | Weight | 91% |
|  | BMI | 94% |
| ***TMJ assessment*** | Jaw Function/Dysfunction | 97% |
|  | Diet | 91% |
|  | Pain onset | 100% |
|  | Pain quality | 100% |
|  | Pain duration | 100% |
|  | Wilkes Staging Classification for Internal Derangement | 78% |
|  | Angle’s Classification | 78% |
|  | Charlson comorbidity Index | 78% |
| ***Clinical assessment – exams*** | Malocclusion | 91% |
|  | General condition of dentition (e.g., tooth wear, decay, etc.) | 84% |
|  | Deviated opening laterality, mm from midline | 91% |
|  | Maximum Interincisal Opening | 94% |
|  | Range of Motion (ROM) | 91% |
|  | Muscle palpation of the head and neck muscles (e.g., point tenderness and size of masseter, occipitals, posterior cervical, sternocleidomastoid (SCM), temporalis, trapezius) | 94% |
|  | Examination of temporalis tendon | 78% |
|  | Pressure pain threshold | 75% |
|  | Facial asymmetry | 84% |
|  | Other, specify | 78% |
| ***Clinical assessment-based diagnosis (Y/N)*** | Diagnosis based on clinical assessment | 97% |
| ***Clinician information*** | Clinician Identifier | 88% |
|  | Type of Clinician | 97% |
|  | Facility Identifier | 84% |
| ***Laboratory findings*** | Antinuclear Antibodies (ANA) Test | 78% |
|  | Basic Metabolic Panel (BMP) | 66% |
|  | C-Reactive Protein (CRP) Test | 78% |
|  | Complete Blood Count (CBC) Test | 75% |
|  | Metal-LTT Blood Test | 72% |
|  | Erythrocyte Sedimentation Rate (ESR) Test | 69% |
|  | Rheumatoid Factor (RF) Test | 78% |
|  | Rheumatological Lab Tests | 81% |
|  | Other, specify | 63% |
| ***Imaging findings*** | CT Scan with or without contrast | 81% |
|  | Magnetic Resonance Imaging (MRI) findings | 84% |
|  | Frontal cephalometric/Submental vertex findings (symmetry) | 44% |
|  | Panoramic findings | 81% |
|  | Cone beam commuted tomography (CBCT) findings | 72% |
|  | Other Imaging (identify) | 44% |
| ***Clinical assessment - TMJ/D specific diagnostics tests***  ***TMD treatment procedures*** | Clinical assessment - TMJ/D specific diagnostics tests | 50% |
|  | Date of Procedure | 100% |
|  | Procedure Code | 100% |
|  | Interventional Type e.g., (Arthrocentesis, Arthroplasty, TMJ Total Joint Replacement, Coronoidectomy, Orthognathic Surgery with TMJ TJR, Joint Replacement for Tumor or Trauma, Others with Vascular/Bone Fibula Grafts) | 97% |
|  | Interventional Site (body location) | 100% |
|  | Procedure Status (e.g., Completed, Treatment Aborted, Incomplete) | 94% |
|  | Procedure Urgency (e.g., Elective, Urgent, Emergency) | 84% |
|  | Facility (location) | 88% |
|  | Surgeon(s) performing the procedure | 91% |
|  | Length of Procedure | 94% |
| ***Arthrocentesis procedures*** | Anesthesia Type | 88% |
|  | Prep and Drape | 72% |
|  | Device Details - Single Needle Gauge | 81% |
|  | Device Details - Double Needle Gauge | 84% |
|  | Device Details - Small Diameter Arthroscopy (1MM) Type | 81% |
|  | Procedure Details - Complications | 97% |
|  | Procedure Details - Additional Procedures Needed | 94% |
| ***Arthroscopy procedures*** | Anesthesia Type | 88% |
|  | Prep and Drape | 72% |
|  | Single Portal | 88% |
|  | Double Portal | 88% |
|  | Triple Portal | 88% |
|  | Level 1 | 88% |
|  | Level 2 | 88% |
|  | Level 3 | 88% |
|  | Biopsy Required | 91% |
|  | Fluid Analysis | 88% |
|  | Lavage Type | 88% |
|  | Lavage Amount | 84% |
|  | Medication Type | 97% |
|  | Medication Amount | 91% |
|  | Complications | 97% |
|  | Use of Laser | 94% |
|  | Additional Procedures Needed | 88% |
| ***Arthroplasty procedure*** | Anesthesia Type | 88% |
|  | Prep and Drape | 75% |
|  | Incision Type | 91% |
|  | Soft Tissue Debridement | 94% |
|  | Disc Displacement Reduction And Fixation | 94% |
|  | Meniscectomy | 94% |
|  | Hard Tissue Reduction And Contouring: Condyle | 94% |
|  | Hard Tissue Reduction And Contouring Fossa/Eminence | 94% |
|  | Temporalis Flap Interposition | 94% |
|  | Fat Graft Interposition | 94% |
|  | Spacer Synthetic Temporary Material | 94% |
|  | Complications | 94% |
|  | Additional Procedures Needed | 94% |
| ***TMJ total joint replacement (TJR) procedures*** | Anesthesia | 84% |
|  | Prep And Drape | 75% |
|  | UDI Stock TMJ TJR | 94% |
|  | UDI Custom TMJ TJR | 94% |
|  | Incisions Type | 88% |
|  | Incisions Position | 84% |
|  | Fat Graft | 97% |
|  | Bone Cement | 88% |
|  | Fossa Component/Screws | 94% |
|  | Mandible Component/Screws | 94% |
|  | Complications | 97% |
|  | Improper Fit Of Fossa Component, Mandible Component, Fossa Screws, Mandibular Screws. Use Of Emergency Screws. | 94% |
|  | Operation Changed From Custom To Stock TJR | 91% |
|  | Additional Procedures Needed | 94% |
| ***Coronoidectomy procedure*** | Anesthesia | 84% |
|  | Prep And Drape | 75% |
|  | Extra Oral | 94% |
|  | Intra-Oral | 94% |
|  | With TJR | 97% |
|  | Removal Of Coronoid Process In Toto | 97% |
|  | Leave Part Of The Coronoid Process Attached To Temporalis Muscle | 97% |
|  | Complications | 97% |
|  | Additional Procedures Needed | 94% |
| ***Orthognathic surgery with TMJ TJR procedure*** | Anesthesia | 84% |
|  | Prep And Drape | 75% |
|  | Maxilla Surgery | 97% |
|  | Mandible Surgery And Splints | 97% |
|  | Maxilla And Mandible Surgery | 97% |
|  | Turbinectomies | 91% |
|  | Total Joint Replacement | 100% |
|  | Complications: Bleeding, Control Of Bleeding | 94% |
|  | Additional Procedures Needed | 94% |
| ***Joint replacement for tumor, trauma, others with vascular/bone fibula grafts procedure*** | Anesthesia | 84% |
|  | Prep And Drape | 75% |
|  | Resection Of Tumor Or Bony Fracture Per Protocol | 97% |
|  | Harvest Of Vascular/Bony Fibula Graft | 91% |
|  | Reconstruction Of TMJ/Fossa/Mandible With Fibula Graft | 94% |
|  | Fibula Grafts Complications | 94% |
|  | Additional Procedures Needed | 94% |
| ***Intraoral Vertical Ramus Osteotomy And Intermaxillary Fixation procedures*** | Anesthesia | 84% |
|  | Prep And Drape | 75% |
|  | Intra-Oral Osteotomy Of The Mandibular Ramus From Sigmoid Notch To The Angle Of The Mandible Right, Left Or Both | 97% |
|  | Intermaxillary Fixation | 97% |
|  | Complications: Bleeding, Control Of Bleeding, Other | 94% |
|  | Additional Procedures Needed | 94% |
| ***TMD device details and device characteristics*** | Device UDI | 100% |
|  | Device type | 100% |
|  | Device class | 94% |
|  | DI number | 94% |
|  | Company Name | 97% |
|  | Brand Name | 100% |
|  | Model Number | 97% |
|  | Implant material | 97% |
| ***TMD medication*** | Dose | 97% |
|  | Dose Units | 94% |
|  | Code | 81% |
|  | Type Class | 88% |
|  | Start Date | 97% |
|  | End Date | 97% |
| ***Additional Post-operative outcomes*** | Chronic lymphocytic infiltrate present | 81% |
|  | Modified Mirra Score | 69% |
| ***Additional longitudinal follow-up elements*** | Mortality - Date of death | 84% |
|  | Mortality - Cause of death | 78% |
| ***Patient survey tools for TMJ/TMD patients*** | BSI-18 and Pressure Pain Threshold (Pre-Op) | 69% |
|  | Diet Limitation Numeric Rating Scale (NRS) (Pre-Op to 60 months) | 66% |
|  | EuroQoL five dimension (EQ-5D-5L) (Pre-Op to 60 months) | 75% |
|  | Interference with Eating (Visual Analog Scale) | 72% |
|  | Jaw Function limitation scale 8 - (Pre-Op, 3 months, 12 months) | 97% |
|  | Jaw Function Numeric Rating Scale (NRS) (Pre-Op to 60 months) | 69% |
|  | OHIP-TMD (Pre-Op to 60 months) | 84% |
|  | Pain Measurement (Visual Analog Scale) | 75% |
|  | Pain Numeric Rating Scale (NRS) (Pre-Op to 60 months) | 75% |
|  | SF-12 (Pre-Op, 3 months, 12 months) | 78% |

*Note: Shaded cells represent data elements with less than 75% consensus; 32 total clinical participants (note that 2 participants were both clinicians and patients)*
